# Sensory and spinal inhibitory dorsal midline crossing is independent of Robo3

**DOI:** 10.3389/fncir.2015.00036

**Published:** 2015-07-23

**Authors:** John D. Comer, Fong Cheng Pan, Spencer G. Willet, Parthiv Haldipur, Kathleen J. Millen, Christopher V. E. Wright, Julia A. Kaltschmidt

**Affiliations:** ^1^Neuroscience Program, Weill Cornell Graduate School of Medical SciencesNew York, NY, USA; ^2^Developmental Biology Program, Sloan-Kettering InstituteNew York, NY, USA; ^3^Weill Cornell/Rockefeller/Sloan-Kettering Tri-Institutional MD-PhD ProgramNew York, NY, USA; ^4^Vanderbilt University Program in Developmental Biology, Department of Cell and Developmental Biology, Vanderbilt Center for Stem Cell Biology, Vanderbilt University Medical CenterNashville, TN, USA; ^5^Seattle Children’s Research Institute, Center for Integrative Brain ResearchSeattle, WA, USA; ^6^Department of Pediatrics, Genetics Division, University of WashingtonSeattle, WA, USA

**Keywords:** commissural neuron circuitry, spinal cord, dorsal midline, midline crossing, Robo1, Robo2, Robo3, axon guidance

## Abstract

Commissural neurons project across the midline at all levels of the central nervous system (CNS), providing bilateral communication critical for the coordination of motor activity and sensory perception. Midline crossing at the spinal ventral midline has been extensively studied and has revealed that multiple developmental lineages contribute to this commissural neuron population. Ventral midline crossing occurs in a manner dependent on Robo3 regulation of Robo/Slit signaling and the ventral commissure is absent in the spinal cord and hindbrain of *Robo3* mutants. Midline crossing in the spinal cord is not limited to the ventral midline, however. While prior anatomical studies provide evidence that commissural axons also cross the midline dorsally, little is known of the genetic and molecular properties of dorsally-crossing neurons or of the mechanisms that regulate dorsal midline crossing. In this study, we describe a commissural neuron population that crosses the spinal dorsal midline during the last quarter of embryogenesis in discrete fiber bundles present throughout the rostrocaudal extent of the spinal cord. Using immunohistochemistry, neurotracing, and mouse genetics, we show that this commissural neuron population includes spinal inhibitory neurons and sensory nociceptors. While the floor plate and roof plate are dispensable for dorsal midline crossing, we show that this population depends on Robo/Slit signaling yet crosses the dorsal midline in a Robo3-independent manner. The dorsally-crossing commissural neuron population we describe suggests a substrate circuitry for pain processing in the dorsal spinal cord.

## Introduction

Bilateral neuronal communication is present at all levels of the central nervous system (CNS) and underlies a diverse array of neuronal functions, including the coordination of motor activity (Butt and Kiehn, 2003; Mueller et al., 2009), sensory perception (Bermingham et al., 2001; Li and Ebner, 2006) and interhemispheric cortical processing (Bloom and Hynd, 2005). In the spinal cord, studies of bilateral communication have focused largely on the ventral horn, where genetically identified commissural neuron populations cross at the ventral midline and provide the left-right coordination of motor activity essential for locomotion (Lanuza et al., 2004; Zhang et al., 2008). The axon guidance mechanisms that coordinate ventral midline crossing have been studied extensively. This work has identified roles for attractive and repulsive guidance cues, as well as for the regulation of commissural axon responsiveness to these cues (Tessier-Lavigne and Goodman, 1996; Dickson and Zou, 2010). Anatomical studies have demonstrated commissural projections that also cross the dorsal midline, but less is known regarding the origins and molecular identities of dorsally-crossing commissural neurons (DCNs) or the guidance cues that control dorsal midline crossing.

Ramón y Cajal (1995) provided some of the earliest evidence of axons crossing the dorsal midline, demonstrating that these axons originate within the dorsolateral region of the spinal cord and cross the dorsal midline in three distinct bundles distributed between the ventral aspect of the dorsal funiculus and the central canal. A more recent study of the embryonic rat spinal cord similarly reported three distinct bundles of fibers at the dorsal midline (Orlino et al., 2000). Dextran neutrotracing in the adult rat also identified a neuronal population whose cell bodies are restricted to the dorsolateral spinal cord and project to the same region in the contralateral dorsal horn, with terminals expressing synaptic proteins associated with inhibitory neurons (Petkó and Antal, 2000; Petkó et al., 2004). While these data support a CNS origin for some DCNs, multiple neurotracing studies in the embryonic and postnatal rat have demonstrated that sensory fibers also cross the dorsal midline (Smith, 1983; Snider et al., 1992; Mirnics and Koerber, 1995).

Cytochemical studies of the spinal cord following unilateral nerve manipulations also suggest bilateral connectivity in the dorsal spinal cord. Numerous studies in rats have shown that unilateral manipulations of peripheral nerves result in contralateral changes in gene expression in the dorsal horn (Koltzenburg et al., 1999). These changes include decreased gamma-aminobutyric acid (GABA) and reduced expression of the GABA transporter GAT-1 (Ibuki et al., 1997; Miletic et al., 2003), supporting the presence of inhibitory DCNs, and also include increases in sensory neuropeptide expression (Zhang et al., 1996), supporting the contribution of sensory neuronal populations. While sensory neuron neuropeptide expression is variably distributed among sensory neurons, studies comparing conduction velocity and neuropeptide immunoreactivity suggest that the neuropeptides may be preferentially expressed in C and Aδ fibers, which function in nociception and mechanosensation (Lawson, 1995; Lawson et al., 1997). Electrophysiological studies also provide evidence of bilateral dorsal spinal cord connectivity in pain pathways. In the decerebrate spinal rat, the activity of dorsal horn neurons responsive to nociceptive stimuli was found to be depressed by noxious stimuli applied to the contralateral limb and tail, indicating a bilateral connectivity supported by spinal commissural neurons or a direct sensory relay of the nociceptive response (Fitzgerald, 1982). However, while these studies hint at the properties of bilateral connectivity within the dorsal spinal cord, the molecular and genetic identities of DCNs remain unclear, and our understanding of the development of this bilateral connectivity remains limited.

Mouse genetics has revealed the developmental lineages and axon guidance mechanisms used by commissural neurons that cross at the ventral midline, supporting the use of mice for the study of the developmental and molecular properties of the DCN population (Kaprielian et al., 2000; Dickson and Zou, 2010). However, evidence of dorsal midline crossing in the mouse spinal cord has been primarily limited to sensory neuron populations that have been shown to project contralaterally during later stages of development (Ozaki and Snider, 1997), leaving contributions from spinal populations unknown. Further, while commissural axon navigation of the ventral midline has been extensively studied, little is known regarding this critical developmental process at the dorsal midline.

In the spinal cord and hindbrain, attractive and repulsive guidance cues derived from the floor plate direct commissural axons across the ventral midline (Tessier-Lavigne and Goodman, 1996). Commissural axons are attracted to the ventral midline by the attractive guidance cue Netrin-1 (Kennedy et al., 1994; Serafini et al., 1994, 1996; Keino-Masu et al., 1996) and are then expelled to enter the contralateral CNS by repulsive cues belonging to the Slit family (Brose et al., 1999), which bind the Robo receptors Robo1 and Robo2 (Long et al., 2004). In the contralateral spinal cord, Robo1 and Robo2 have been shown to function in the sorting of post-crossing commissural axons to the ventral and lateral funiculi (Jaworski et al., 2010). Robo1 and Robo2 protein are also expressed on pre-crossing commissural axons (Long et al., 2004; Sabatier et al., 2004), requiring regulation of commissural axon responsiveness to Slit-mediated repulsion to permit entering and crossing at the ventral midline. Alternative splicing of the divergent Robo family member Robo3 has been shown to produce two spatially segregated isoforms: Robo3.1, which suppresses Slit-mediated repulsion of pre-crossing commissural axons, and Robo3.2, which favors Slit repulsion of post-crossing commissural axons and prevents inappropriate recrossing of the midline (Chen et al., 2008). *Robo3* loss of function, which deletes the activity of both Robo3 isoforms, has been shown to prevent crossing at the ventral midline, supporting the view that Robo3 is required for commissure formation in the spinal cord and hindbrain (Marillat et al., 2004; Sabatier et al., 2004; Chen et al., 2008). Whether similar mechanisms regulate crossing at the dorsal midline, however, remains unclear.

Here, we report a commissural neuron population in the developing mouse spinal cord that crosses at the dorsal midline. Using mouse genetics, we find that this population of DCNs is comprised of spinal inhibitory neurons arising from a dorsal neuronal lineage and sensory nociceptors. Similar to ventrally-crossing commissural neurons, we find that DCNs require Robo1/Robo2 and Slit signaling to traverse the midline; however, they cross the midline independently of Robo3.

## Materials and Methods

### Mouse Strains

The *Ptf1a* null allele (*Ptf1a*^Null^) was generated from a *Ptf1a* floxed allele (*Ptf1a*^Flox^), which will be described in full detail in a future publication. *Ptf1a*^Flox^ was constructed such that loxP sites were located upstream and downstream of the gene. Crossing *Ptf1a*^Flox^ to a germline Cre-driver (*E2A*^Cre^; Lakso et al., 1996) caused the deletion of the entire Ptf1a gene including all protein-coding sequences. Please contact C.V.E. Wright for full details.

The following additional mouse strains were used in this study: *Ptf1a^Cre/+^* (Kawaguchi et al., 2002), *Ptf1a*^CreER/+^(Pan et al., 2013), *Advillin*^Cre/+^ (Zhou et al., 2010; da Silva et al., 2011), *R26*^CAG−lox−STOP−tdTomato/+^ (Jackson, Ai14; referred to as *R26*^lox−tdT/+^), *Robo1/2*^+/−^ (Chen et al., 2008), *Robo3*^+/−^ (Sabatier et al., 2004), *Gli2*^lzki/+^ (Bai and Joyner, 2001), *Nfia*^+/−^ (das Neves et al., 1999), and *Lmx1a*^dr−J/+^ (*dreher*; Millonig et al., 2000).

At least three embryos were analyzed for every genotype. Experiments conform to the regulatory standards of the Institutional Animal Care and Use Committee of Memorial Sloan-Kettering Cancer Center.

### Histochemistry

Immunohistochemistry and *in situ* hybridization on 30 μm and 12 μm thick cryostat sections, respectively, were performed as previously described (Arber et al., 1999; Betley et al., 2009). The following antibodies were used in 0.3% phosphate buffer triton (PBT); 1% bovine serum albumin (BSA); 0.3% Triton-X in phosphate buffer saline (PBS): rat anti-L1 Cell Adhesion Molecule (anti-L1CAM; 1:400; Millipore), rabbit anti-red fluorescent protein (anti-RFP; 1:1000; Rockland), guinea pig anti-RFP (1:2000; Betley et al., 2013), mouse anti-glutamic acid decarboxylase (anti-GAD-6; 1:1000; Abcam), rabbit anti-tryosine kinase receptor A (anti-TrkA; 1:5000; generously provided by L. Reichardt; Huang et al., 1999), chicken anti-TrkB (1:500; generously provided by L. Reichardt; Huang et al., 1999), chicken anti-Parvalbumin (Pv; 1:10,000; generously provided by S. Brenner-Morton and T. Jessell; de Nooij et al., 2013), rabbit anti-S100β (1:1000; Dako), and fluorophore-conjugated secondary antibodies (Jackson Labs and Molecular Probes). Anti-sense *in situ* probes were generated from mouse E12.5 spinal cord cDNA using polymerase chain reaction (PCR) amplification. The following primers were used: *Robo1* (forward primer (FP): CAGGCAACAACCACAATGAC, reverse primer (RP): AGTGGGGCCTCTTTCATCTT), *Robo2* (FP: AAGGGGAACAACGCCTTACT, RP: GCTCCGGACACGTAACCTAA), *Robo3* (FP: AAGGATTCCGTGTGTCTTGG, RP: GAGTTCTTTGCGCTGCTTCT), *Slit1* (FP: TGTTGCAGCTGATGGAGAAC, RP: GTGGGATGGATTTGATACCG), *Slit2* (FP: AACAACAACCCACCTTCCAG, RP: CCCAGAAGAGCAAAGCAAAG), *Slit3* (FP: ACTGGGGACTCCTACGTGTG, RP: CACAACACAAAACAAAACTTGG), and *Netrin-1* (FP: CTGGGTGGAGTTCACCATCT, RP: ACAAAGAAGGCAGCCAGAAA).

### Lipophilic Dye Neurotracing

For DCN neurotracing, embryos were dissected, the spinal cord was exposed via dorsal laminectomy, fixed in 4% paraformaldehyde (PFA) for 2 h, and washed in PBS. For spinal DCN neurotracing, DiI crystals (Molecular Probes) were placed in the dorsolateral region of the spinal cord using glass micropipettes (Renier et al., 2010). For supraspinal neurotracing, the skull was removed, and DiI crystals were placed into the caudal hindbrain. For sensory DCN neurotracing, DiI crystals were placed in dorsal root ganglia and the ventral roots were cut. Tissue samples were placed in 4% PFA at 37°C for up to 5 weeks for DiI diffusion. Samples were then washed with PBS and embedded in 4–6% agarose for vibratome sectioning (200 μm). Sections were mounted with PBS for confocal microscopy.

## Results

### DCNs Cross the Dorsal Midline During Late Embryogenesis

**Figure 1:**
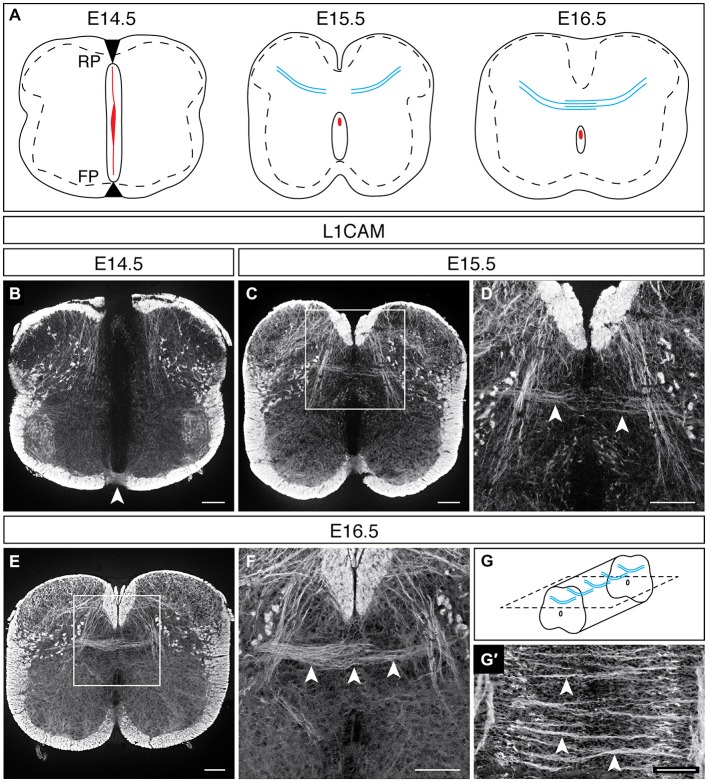
**DCN midline crossing during embryonic development. (A)** At E14.5, the spinal midline is comprised of the roof plate (RP), the neural canal (red), and the floor plate (FP). By E15.5, the neural canal reduces in size, and DCNs are approaching the dorsal midline (blue). At E16.5, DCN axons have crossed the dorsal midline. **(B)** At E14.5, L1CAM^ON^ commissural axons cross at the ventral midline (arrowhead; Dodd et al., 1988); however, no L1CAM^ON^ DCN axons are present. **(C,D)** At E15.5, L1CAM^ON^ DCN axons are approaching the dorsal midline [arrowheads in **(D)**]. **(D)** corresponds to boxed region in **(C). (E,F)** By E16.5, L1CAM^ON^ DCN axons have crossed the dorsal midline [arrowheads in **(F)**]. **(F)** corresponds to boxed region in **(E). (G,G′)** Longitudinally, discrete bundles of L1CAM^ON^ DCN axons are present [arrowheads in **(G′)**]. Schematic of longitudinal sections in **(G)**. Scale bars: 100 μm.

In rat embryos, L1CAM immunohistochemical studies demonstrated that DCNs cross the dorsal midline during the last quarter of embryogenesis (Orlino et al., 2000), a developmental period during which neurogenesis is ending and the central canal is taking on its mature form (Sevc et al., 2009). To define the embryonic stage at which DCNs in the developing mouse spinal cord begin to cross the dorsal midline, we turned our attention to mouse embryonic stages that resemble this rat developmental period. BrdU and autoradiographic analyses have shown that neurogenesis in the mouse spinal cord ends by embryonic day (E) 14.5 (Nornes and Carry, 1978; Gross et al., 2002; Müller et al., 2002). Moreover, by E15.5, the ventricular zone recedes and the dorsal aspect of the neural canal fuses (Sturrock, 1981), providing a putative pathway for DCN axons to cross to the contralateral dorsal horn. Thus, to choose the earliest developmental stage at which DCNs may be detected, we selected E14.5 and used L1CAM immunohistochemistry to visualize DCN axons. At E14.5, the neural canal still occupies nearly the entirety of the midline along the dorsoventral axis, its dorsal and ventral aspects apposed to the putative roof plate and floor plate, respectively (Figure 1A; Sturrock, 1981). In addition to its expression in the dorsal and ventral funiculi, L1CAM is present in commissural axons that cross at the ventral midline (Dodd et al., 1988), as well as in sensory afferents projecting into the dorsal horn (Figure 1B). However, we did not detect L1CAM-expressing (L1CAM^ON^) axons approaching the dorsal midline at this embryonic stage. By E15.5, the central canal is nearing its mature form, and we detected L1CAM^ON^ DCN axons approaching the dorsal midline, with some axons beginning to enter the contralateral side (Figures 1A,C,D). By E16.5, we detected L1CAM^ON^ DCN axons clearly crossing the midline to the contralateral dorsal horn (Figures 1A,E,F). In longitudinal sections of E16.5 embryos, we found that L1CAM^ON^ DCN axons cross in discrete bundles that are present throughout the rostrocaudal extent of the spinal cord (Figures 1G,G′; Orlino et al., 2000). Further, at this stage, L1CAM^ON^ longitudinal tracts are present at the dorsoventral level of the dorsal commissure, raising the possibility that DCNs project some distance rostrocaudally (Figure 1G′), as has been reported in the adult rat (Petkó and Antal, 2000). Together, these results show that DCN midline crossing in the developing mouse spinal cord occurs during the last quarter of embryogenesis.

### Spinal and Sensory Neurons Contribute to the DCN Population

**Figure 2:**
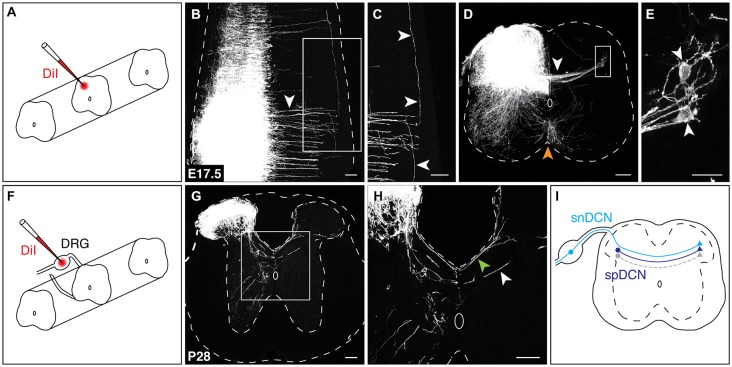
**Spinal and sensory neurons contribute to the DCN population. (A)** For spinal DCN neurotracing, DiI crystals were placed in the dorsolateral region of the spinal cord. **(B,C)** At E17.5, DiI-labeled DCN axons cross in discrete bundles [arrowhead in **(B)**] and project longitudinally in both the rostral and caudal directions [arrowheads in **(C)**]. **(D,E)** In transverse sections, both DCN [white arrowhead in **(D)**] and commissural axons at the ventral midline (orange arrowhead) are labeled. spDCN cell bodies are labeled in the contralateral dorsolateral region of the spinal cord [white arrowheads in **(E)**]. **(E)** corresponds to boxed region in **(D). (F)** For snDCN neurotracing, DiI crystals were placed in DRG. **(G,H)** At P28, snDCNs cross the dorsal midline in two bundles, a posterior bundle (green arrowhead) and centrally at the dorsal midline (white arrowhead). snDCNs terminate within the dorsomedial and dorsolateral regions of the contralateral spinal cord. Sections from caudal thoracic and rostral lumbar spinal cord. **(I)** Summary of DCN populations. Predicted gray population indicates that DCNs are heterogeneous (see “Discussion” Section). Scale bars: 100 μm **(B–D,G,H)**; 25 μm **(E)**.

Neurotracing studies in adult rat have identified neurons in the spinal cord that give rise to fibers crossing at the dorsal midline (Petkó and Antal, 2000); however, it is unknown if a population of spinal DCNs (spDCNs) is similarly present in the developing mouse spinal cord. To test for the presence of spDCNs, we applied the lipophilic dye DiI to the dorsolateral region of the spinal cord of E17.5 embryos, a developmental stage chosen to improve the likelihood that DCN axons had extended sufficiently far into the contralateral dorsal horn (Figure 2A). DiI neurotracing revealed DCN axons crossing the dorsal midline, and, similar to our L1CAM analysis, showed that they cross in discrete bundles (Figure 2B). Further, retrograde DiI diffusion revealed labeled spDCN cell bodies within the contralateral dorsal horn (Figures 2D,E). Moreover, we also found that DiI-labeled DCN axons projected longitudinally in both the rostral and caudal directions (Figure 2C), suggesting that DCNs may provide intersegmental connectivity.

To test whether sensory neurons contribute to the DCN population, we next applied DiI crystals to postnatal dorsal root ganglia (DRG; Figure 2F). DiI neurotracing showed sensory DCN (snDCN) axons crossing within the central region of the dorsal midline and along the perimeter of the dorsal funiculus (Figures 2G,H), in agreement with previous studies (Smith, 1983; Mirnics and Koerber, 1995; Ozaki and Snider, 1997). These snDCNs project contralaterally to the medial or lateral dorsal horn, the latter coinciding with the location of spDCN cell bodies (Figure 2G). To test whether DCNs also project supraspinally, we placed DiI crystals into the caudal hindbrain of E16.5 embryos. While we frequently detected retrogradely-labeled cells that cross at the ventral midline, we never observed any retrogradely-labeled cells that cross at the dorsal midline, suggesting that DCNs do not project supraspinally (data not shown). Together, these results show that a spinal and sensory component contribute to the DCN population (Figure 2I).

### Spinal DCNs Belong to the Ptf1a-Expressing Lineage and Sensory DCNs are Nociceptors

**Figure 3:**
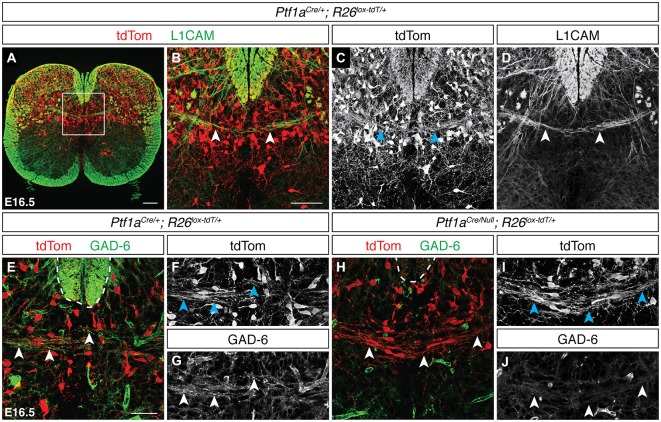
**Neurons of the Ptf1a-expressing lineage contribute to the spinal DCN population. (A–D)** At E16.5, tdTom^ON^/L1CAM^ON^ spDCN axons cross the dorsal midline in *Ptf1a*^Cre/+^; R26^lox−tdT/+^ spinal cord [arrowheads in **(B–D)**]. **(B–D)** correspond to boxed region in **(A). (E–G)** At E16.5, tdTom^ON^/GAD-6^ON^ spDCN axons are present at the dorsal midline in *Ptf1a*^Cre/+^; R26^lox−tdT/+^ spinal cord [arrowheads in **(E–G)**]. Dotted line indicates the dorsal funiculus. **(H–J)** GAD-6 labeling is lost in spDCN axons in E16.5 *Ptf1a*^Cre/Null^; R26^lox−tdT/+^ spinal cord [arrowheads in **(H–J)**]. Dotted line indicates the dorsal funiculus. Scale bars: 100 μm **(A)**; 50 μm **(B–J)**.

Previous electrophysiological studies have described contralateral inhibitory signaling in the dorsal spinal cord (Fitzgerald, 1982), suggesting the presence of inhibitory spDCNs. Inhibitory neurons in the dorsal spinal cord arise from the dI4 and dIL^A^ lineages and specifically express the basic helix-loop-helix (bHLH) transcription factor Ptf1a (Glasgow et al., 2005). To test whether these lineages contribute to the DCN population, we used *Ptf1a*^Cre/+^; R26^lox−tdT/+^ mice, which restrict tdTom reporter expression to dI4 and dIL^A^ neurons (Figure 3A). At E16.5, we found tdTom^ON^/L1CAM^ON^ projections crossing the dorsal midline in discrete bundles (Figures 3B–D; data not shown), supporting the view that Ptf1a-expressing neurons contribute to the DCN population. To show that these Ptf1a-expressing spDCNs are GABAergic, we used the GAD-6 monoclonal antibody, which binds the GABA-producing enzyme GAD65 (Kaufman et al., 1991), and detected GAD-6^ON^/tdTom^ON^ DCN axons crossing at the dorsal midline (Figures 3E–G). To confirm that these inhibitory GAD-6^ON^ DCNs require Ptf1a and thus arise from the dI4 or dIL^A^ lineages, we analyzed *Ptf1a*^Cre/Null^; R26^lox−tdT/+^ mutant embryos, where *Ptf1a* expression is lost and dorsal horn inhibitory neurons are misspecified as dI5 or dIL^B^ excitatory neurons (Glasgow et al., 2005). While we still observed tdTom^ON^ spDCN axons, we found that the GAD-6 DCN labeling was lost, confirming that GABAergic inhibitory spDCNs belong to the Ptf1a-expressing lineage (Figures 3H–J; *N* = 3).

**Figure 4:**
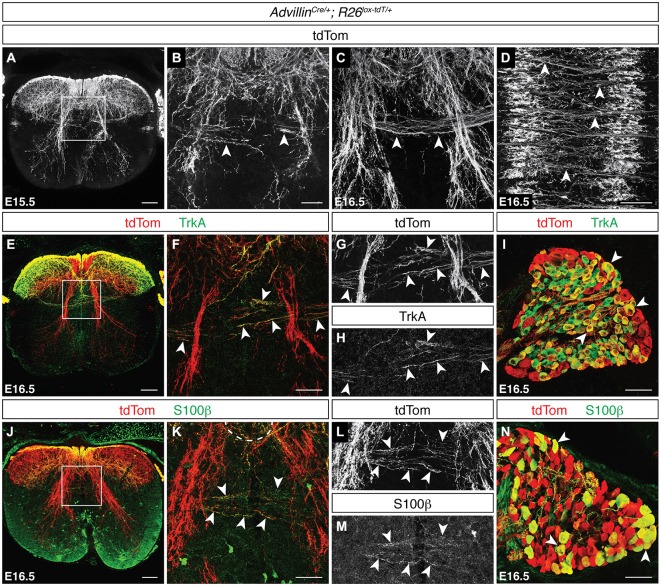
**Sensory DCNs belong to a S100β^ON^ population of nociceptors. (A–D)** At E15.5 and E16.5, tdTom^ON^ snDCN axons in *Advillin*^Cre/+^; R26^lox−tdT/+^ spinal cord [arrowheads in **(B,C)**] cross the dorsal midline in discrete bundles [arrowheads in **(D)**]. **(E–I)** tdTom^ON^ snDCN axons co-label with TrkA [arrowheads in **(F–H)**], which is widely expressed in DRG (**I**; Mu et al., 1993). **(F–H)** correspond to boxed region in **(E). (J–N)** tdTom^ON^ snDCN axons double label with S100β in *Advillin*^Cre/+^; R26^lox−tdT/+^ embryos at E16.5 [arrowheads in **(K–M)**]. tdTom^ON^/S100β^ON^ cell bodies are present in DRG [arrowheads in **(N)**]. **(K–M)** correspond to boxed region in **(J)**. Scale bars: 100 μm **(A,D,E,J)**; 50 μm **(B,C,F–I,K–N)**.

To analyze the development of snDCNs, we used *Advillin*^Cre/+^; R26^lox−tdT/+^ mice, in which neurons of neural crest origin express tdTom (Hasegawa et al., 2007). We detected tdTom^ON^ snDCNs at the dorsal midline at E15.5, which clearly cross to the contralateral dorsal horn by E16.5 (Figures 4A–C). Additionally, we found that these tdTom^ON^ contralateral projections cross in discrete bundles throughout the rostrocaudal extent of the spinal cord and co-label with L1CAM (Figure 4D, data not shown). To functionally classify snDCNs, we used immunuhistochemistry at E16.5 for TrkA, which is primarily expressed by nocioceptive sensory neurons (Snider and McMahon, 1998; Fang et al., 2005), and the calcium-binding protein Pv, a proprioceptive sensory neuron marker (Mu et al., 1993; Honda, 1995). Pv^ON^ fibers were never observed crossing at the dorsal midline (data not shown). TrkA^ON^ sensory axons, however, are present at the dorsal midline and co-label with tdTom^ON^ snDCN axons in *Advillin*^Cre/+^; R26^lox−tdT/+^ embryos, suggesting that snDCNs belong to the nociceptive class of sensory neurons (Figures 4E–I). A screen for additional DCN axon markers found that the calcium-binding protein S100β also labels DCN axons at the dorsal midline. While primarily thought to be a glial cell marker, S100β is also expressed by neurons in the brain (Friend et al., 1992), and a transgenic mouse line expressing enhanced green fluorescent protein (EGFP) under the control of the S100β promoter has demonstrated EGFP^ON^ sensory neurons (Vives et al., 2003), suggesting that sensory neurons also express S100β. In E16.5 *Advillin*^Cre/+^; R26^lox−tdT/+^ embryos, we found that S100β is present in the dorsal horn and co-labels with tdTom^ON^ snDCN fibers at the dorsal midline (Figures 4J–N), indicating that snDCNs belong to a S100β^ON^ subpopulation of nociceptive sensory neurons.

### DCNs Utilize Robo1/2 but not Robo3 for Dorsal Midline Crossing

**Figure 5:**
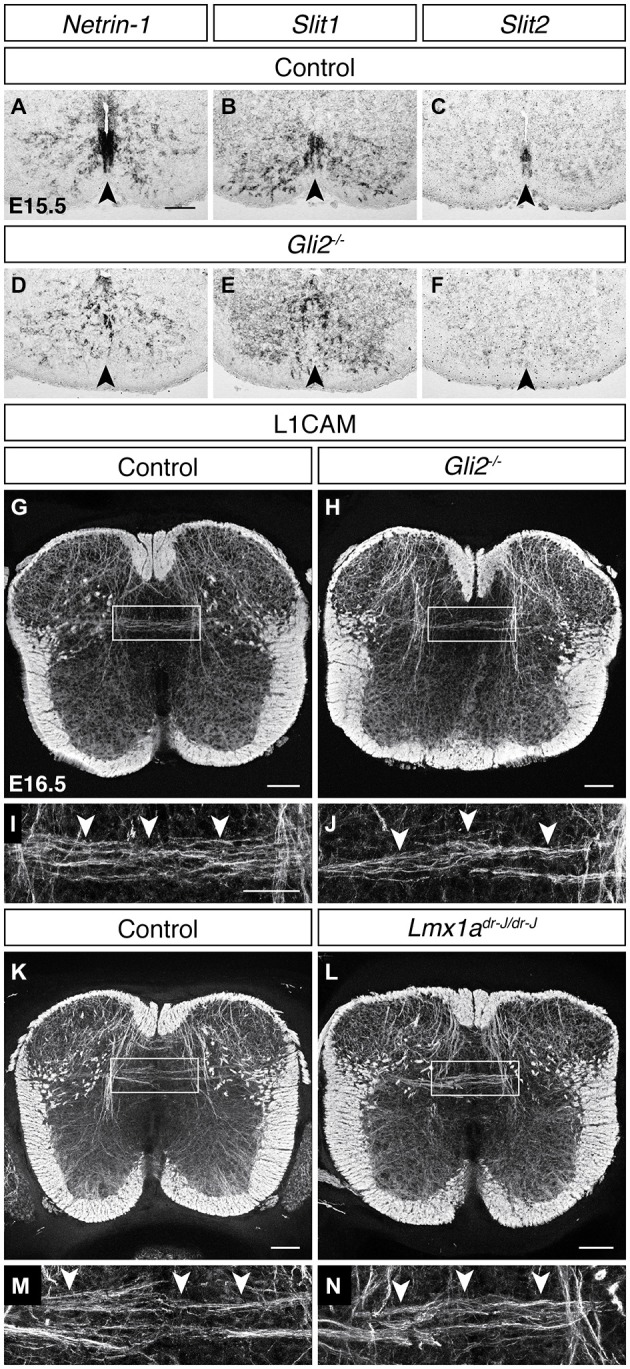
**DCN midline crossing does not depend on floor plate-derived guidance cues or on the roof plate. (A–F)** At E15.5, guidance cue transcript expression of *Netrin-1* **(A)**, *Slit1* **(B)** and *Slit2* **(C)** at the ventral midline is lost or disorganized in the *Gli2* mutant (**D–F**; Matise et al., 1999). **(G–J)** L1CAM^ON^ DCN axons persist in E16.5 *Gli2* mutant spinal cords, compared to control. **(I)** corresponds to boxed region in **(G). (J)** corresponds to boxed region in **(H). (K–N)** L1CAM^ON^ DCN axons persist in E16.5 *Lmx1a (dreher)* mutant spinal cords, compared to control. **(M)** corresponds to boxed region in **(K). (N)** corresponds to boxed region in **(L)**. Scale bars: 100 μm **(A–H,K,L)**; 50 μm **(I,J,M,N)**.

The floor plate provides both attractive and repulsive guidance cues and has been shown to be a critical organizing structure for commissural neuron midline crossing (Tessier-Lavigne and Goodman, 1996), raising the possibility that floor plate-derived guidance cues may also play a role in midline crossing by DCN axons. To test this, we first assessed the transcript expression of the attractive guidance cue *Netrin-1* at E15.5 and found that it is strongly expressed at the ventral midline (Figure 5A). We next assessed the expression of the repulsive guidance cues *Slit1*, *Slit2*, and *Slit3* at this age and found that both *Slit1* and *Slit2* are expressed at the ventral midline while *Slit3* transcript is undetectable (Figures 5B,C; data not shown). Together, these results show that floor plate-derived guidance cues are present during DCN midline crossing. To test if DCN midline crossing depends on these floor plate derived guidance cues, we analyzed *Gli2* mutants in which the floor plate does not form and ventral midline guidance cue expression is perturbed (Matise et al., 1998, 1999). In E15.5 *Gli2* mutant mice, transcript expression of *Netrin-1* (Matise et al., 1999), *Slit1*, and *Slit2* was either lost or disorganized at the ventral midline (Figures 5D–F; *N* = 4). We used immunohistochemistry for L1CAM to visualize DCN axons in E16.5 *Gli2* mutant embryos and found that L1CAM^ON^ DCN axons continue to cross at the dorsal midline (Figures 5G–J; *N* = 3), demonstrating that DCN midline crossing does not depend on floor plate-derived guidance cues or on the floor plate itself.

Because DCN midline crossing does not commence until after dorsal midline fusion, we also considered a role of the roof plate in DCN midline crossing. Previous studies have shown that the roof plate is required for the maturation of the neural canal (Kondrychyn et al., 2013), suggesting that it may play a role in dorsal midline development. In the *dreher* mutant, the roof plate does not form (Millonig et al., 2000), but L1CAM immunohistochemistry revealed that DCN midline crossing persists in this mutant background (Figures 5K–N; *N* = 3), indicating that the roof plate, similarly to the floor plate, is dispensable for DCN contralateral growth.

**Figure 6:**
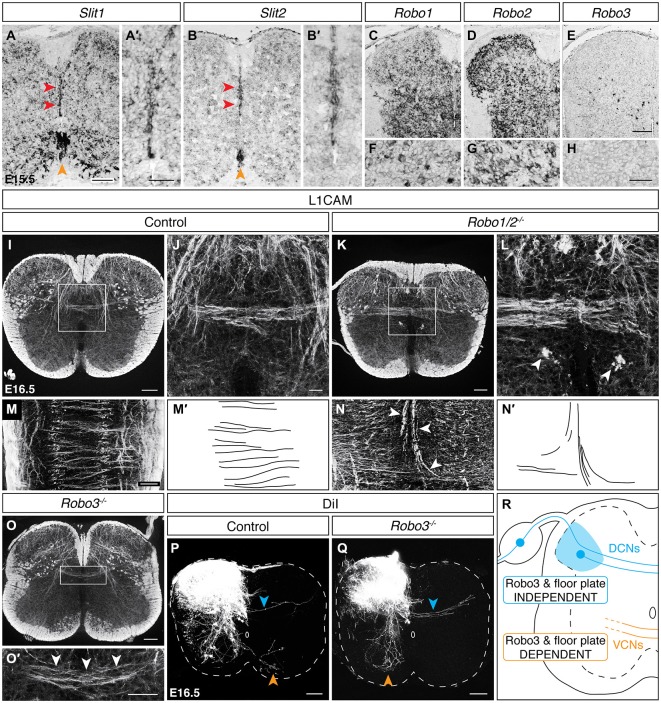
**DCNsrely on Robo/Slit signaling at the dorsal midline but utilize aRobo3-independent midline crossing mechanism. (A–B′)** *Slit1* and* Slit2* are expressed at the dorsal (red arrowheads) and ventral (orange arrowheads) midline in E15.5 spinal cord. The dorsal midline is magnified in **(A′)** and **(B′). (C–H)** At E15.5, *Robo1* and *Robo2* are expressed in dorsolateral spinal cord **(C,D)** and DRG **(F,G)**. *Robo3* is sparsely expressed in the dorsolateral spinal cord **(E)** and is absent from DRG **(H). (I–L)** L1CAM^ON^ ectopic longitudinal funiculi [arrowheads in **(L)**] are present proximally to the dorsal midline in the *Robo1/2* double mutant at E16.5. **(J)** corresponds to the boxed region in **(I). (L)** corresponds to boxed region in **(K). (M–N′)** In longitudinal sections, L1CAM^ON^ misguided axons are present at the dorsoventral level of the dorsal commissure and diverge as they approach the dorsal midline, likely comprising the ectopic longitudinal funiculi present in transverse sections. **(M′)** and **(N′)** represent tracings of control and misguided axons, respectively. **(O,O′)** L1CAM^ON^ DCN axons persist in E16.5 *Robo3* mutant spinal cords. **(O′)** corresponds to boxed region in **(O). (P,Q)** DiI-labeled DCN axons (blue arrowhead) cross the midline in E16.5 *Robo3* mutants, unlike ventrally-crossing commissural axons (orange arrowhead), which fail to cross the midline (**Q**; Sabatier et al., 2004). **(R)** Summary of findings. VCN, ventrally-crossing commissural neurons. Scale bars: 100 μm **(A,B,C–E,I,K,M–O,P,Q)**; 50 μm **(A′,B′,O′,F–H)**; 25 μm **(J,L)**.

We next considered guidance cue transcript expression at the dorsal midline during the period of DCN crossing. At E15.5, while *Netrin-1* and *Slit3* expression is absent along the dorsal midline, both *Slit1* and *Slit2* transcripts are present (Figures 6A-B′; data not shown). We further observed that the *Slit* family receptors *Robo1* and *Robo2* are broadly expressed in the spinal cord, including the dorsolateral region where spDCN cell bodies reside (Figures 6C,D). We similarly found *Robo1* and *Robo2* expression in DRG (Figures 6F,G). To test a requirement of DCNs for Robo1 or Robo2, we assessed axon guidance at the dorsal midline in *Robo1/2* double mutants. In longitudinal sections, we found L1CAM^ON^ axons that did not cross the dorsal midline but rather diverged to follow a longitudinal trajectory and appeared to contribute to ectopic longitudinal funiculi proximal to the dorsal midline (Figures 6I–N′; *N* = 3). While not impacting all dorsally-crossing axons, this phenotype of midline divergence resembles the *Robo1/2* mutant phenotype at the ventral midline where a subset of commissural axons diverges and enters the ventricular zone (Jaworski et al., 2010), as well as the *Robo2* mutant phenotype at the optic chiasm where a subset of retinal ganglion cell (RGC) axons follows a caudal trajectory (Plachez et al., 2008). Together, these results indicate that Robo/Slit signaling is required for proper axon guidance at the dorsal midline.

Previous studies of the ventral midline have shown that regulation of Robo/Slit signaling by Robo3 is required for ventral midline crossing (Sabatier et al., 2004). To assess if Robo3 is similarly required for DCN midline crossing, we first examined *Robo3* transcript expression at E15.5. We found sparse *Robo3* expression in the dorsolateral region of the spinal cord where spDCNs reside (Figure 6E). However, *Robo3* transcript is absent from DRG (Figure 6H), suggesting that at least snDCNs cross the midline independently of Robo3. We next assessed *Robo3* mutants at E16.5 and found that L1CAM^ON^ DCNs still approach and cross the dorsal midline (Figures 6O,O′; *N* = 3). To confirm the persistence of DCN midline crossing, we used DiI neurotracing in E16.5 *Robo3* mutant embryos and found that while ventral midline crossing is lost (Sabatier et al., 2004), DiI-labeled DCN axons continue to cross to the contralateral side (Figures 6P,Q; *N* = 3). Because L1CAM and DiI neurotracing label all DCNs, we also considered the possibility that loss of *Robo3* may differentially impact DCN subpopulations. However, analysis of *Ptf1a*^Cre/+^; R26^lox−tdT/+^ and *Advillin*^Cre/+^; R26^lox−tdT/+^ embryos in a *Robo3* mutant background showed that dorsal midline crossing for sp and snDCNs remains intact (data not shown), suggesting that these DCN subpopulations are not differentially impacted. Together, these results show that DCNs utilize a *Robo3*- and floor plate-independent midline crossing mechanism (Figure 6R).

## Discussion

Peripheral nerve injury and electrophysiological studies have provided evidence supporting the existence of neural connectivity underlying bilateral communication within the dorsal spinal cord (Fitzgerald, 1982; Koltzenburg et al., 1999). While anatomical and developmental studies in the rat (Orlino et al., 2000; Petkó and Antal, 2000; Petkó et al., 2004) have identified neuronal populations that may underlie these cytochemical and physiological observations, no formal study of the development of this dorsal bilateral connectivity or of the properties of the neurons providing this connectivity has been performed in the developing mouse spinal cord. Further, it remains unclear if the commissural populations that provide this connectivity utilize midline crossing mechanisms similar to spinal commissural populations that cross at the ventral midline. Here, we identify a population of DCNs in the developing mouse spinal cord that is composed of spinal inhibitory neurons and sensory nociceptors. Moreover, we show that DCNs do not utilize floor plate-derived axon guidance cues and do not require Robo3 for midline crossing.

### DCNs in the Rodent Spinal Cord

The DCN population that we identify in the developing mouse spinal cord shares both anatomical and cytochemical properties of DCNs reported in the rat. Anatomically, DCNs in both species cross the dorsal midline in discrete bundles found throughout the rostrocaudal extent of the spinal cord (Orlino et al., 2000), and spDCN cell bodies are similarly found in the dorsolateral region of the spinal cord (Petkó and Antal, 2000). Anatomical studies in the rat have also provided evidence that the DCN population comprises a commissural propriospinal network within the lumbar spinal cord (Petkó and Antal, 2000), raising the possibility that the DCN population in the mouse may similarly provide intersegmental connectivity. Cytochemical studies in the rat have reported that a subset of the DCN population is inhibitory. In E19 rat spinal cord, double immunostaining for L1CAM and GAD65 demonstrated a subpopulation of L1CAM^ON^/GAD65^ON^ DCN axons, with L1CAM labeling more axons than GAD65 (Orlino et al., 2000). Also, in adult rat spinal cord, a subset of DCN terminals co-labeled with antibodies against the inhibitory synaptic proteins GAD65, GAD67, and GlyT2 (Petkó et al., 2004). We have similarly found an inhibitory subset of DCNs, identified by both GAD-6 immunohistochemistry and genetic labeling using *Ptf1a*^Cre/+^; R26^lox−tdT/+^ mice, where Ptf1a expression genetically targets inhibitory neurons in the dorsal spinal cord (Glasgow et al., 2005). Additionally, we have found that a subset of nociceptive sensory neurons crosses the dorsal midline and may account for the remaining L1CAM positive fibers reported in the embryonic rat (Orlino et al., 2000), as well as the non-inhibitory dextran-labeled terminals reported in the adult rat (Petkó et al., 2004). However, could there be additional DCN populations? An electrophysiological study in rats proposed the presence of an excitatory commissural population with projections to the contralateral substantia gelatinosa (Fitzgerald, 1983), and our analysis of *Ptf1a*^Cre/Null^; R26^lox−tdT/+^ mutants also allows the possibility of an excitatory DCN population. Loss of Ptf1a has been shown to result in the failure to generate inhibitory neurons in the dorsal horn (Glasgow et al., 2005; Bröhl et al., 2008; Huang et al., 2008). Instead, in the *Ptf1a* mutant, neurons of the dorsal inhibitory dI4 and dIL^A^ lineages are misspecified as the excitatory dI5 and dIL^B^ lineages, respectively (Glasgow et al., 2005). Thus, our finding that tdTom^ON^ DCN axons at the dorsal midline persist in *Ptf1a*^Cre/Null^; R26^lox−tdT/+^ mutants suggests that these excitatory lineages may also contribute to the DCN population. Interestingly, DCN neurotracing in the adult rat has identified a population of non-inhibitory DCN terminals that do not co-label with antibodies against any of the vesicular glutamate transporters (Petkó et al., 2004), suggesting that this predicted population may be an excitatory peptidergic population. The dI5 and dIL^B^ lineages include excitatory peptidergic neurons (Xu et al., 2008), and it will be interesting to consider if such populations indeed contribute to the DCN population. Together, these findings suggest that the DCN population is heterogeneous.

### DCN Axon Guidance and Robo3 Independence

Inappropriate crossing at the dorsal midline has been previously reported following genetic disruptions in Robo/Slit signaling (Ma and Tessier-Lavigne, 2007), as well as loss of function of the ephrin type-A receptor EphA4 (Kullander et al., 2003; Escalante et al., 2013; Paixão et al., 2013), which normally elicits a repulsive response upon binding the ligand ephrinB3 present at the spinal cord midline (Imondi et al., 2000; Kullander et al., 2001). In each of these cases, dorsal midline crossing is likely due to the loss of repulsive axon guidance mechanisms that normally function to restrict ipsilaterally-projecting populations to the same side of the spinal cord. Robo/Slit signaling has also been shown to be required for commissural axon guidance at the midline. In addition to axon stalling at the spinal ventral midline, *Robo1/2* mutants display pathfinding errors in which commissural axons inappropriately turn to invade the ventricular zone (Jaworski et al., 2010). At the optic chiasm in *Robo2* mutants, RGC axons also turn inappropriately to follow a caudal trajectory (Plachez et al., 2008). We describe a similar DCN pathfinding phenotype in *Robo1/2* mutants where DCN axons inappropriately turn to assume a longitudinal trajectory as they approach the dorsal midline. This longitudinal divergence suggests that *Robo1/2* mutant DCN axons may be responding to a midline-derived repellent. While the contact-mediated repulsive ephrins are present at the dorsal midline during the last quarter of embryogenesis (Imondi et al., 2000), the loss of Robo1 and Robo2 is unlikely to affect DCN response to these ligands. Instead, DCNs may be repelled by an as yet unidentified Slit receptor that is unmasked in the *Robo1/2* mutant, a model that has also been proposed in studies of ventrally-crossing commissural neurons in *Robo1/2* mutant spinal cord (Jaworski et al., 2010). The receptor PlexinA1, which binds the repellent Semaphorin3B to support commissural axon exit from the ventral midline (Nawabi et al., 2010; Charoy et al., 2012), has been shown to bind the Slit C-terminal fragment SlitC to elicit commissural axon repulsion (Delloye-Bourgeois et al., 2015). While sensory neuronal PlexinA1 has been shown to be specifically expressed by proprioceptive sensory neurons (Yoshida et al., 2006), excluding PlexinA1 expression by snDCNs, *PlexinA1* transcript is present in the dorsolateral spinal cord during late embryogenesis (Escalante et al., 2013), raising the possibility that PlexinA1 may play a role in spDCN Slit responsiveness and may contribute to the DCN dorsal midline repulsion observed in the *Robo1/2* mutant.

Robo3 expression in ventrally-crossing commissural neurons of the spinal cord and hindbrain suppresses Slit-induced repulsion in pre-crossing commissural axons via Robo1 and Robo2, permitting crossing at the ventral midline (Sabatier et al., 2004). Robo3 has thus been understood to be required for commissure formation in the hindbrain and spinal cord. However, we find that DCNs do not require Robo3 despite a reliance on Robo/Slit signaling for axon guidance at the dorsal midline. Robo3 has recently been shown to collaborate with the Netrin receptor Deleted in Colorectal Cancer (DCC; Keino-Masu et al., 1996) to attract the axons of pontine neurons to the floor plate in the hindbrain (Zelina et al., 2014). Ectopic expression of Robo3 in the dorsal spinal cord has also been reported to elicit ventral outgrowth and ventral midline crossing of dorsal horn neurons (Escalante et al., 2013). These observations suggest that Robo3 function may be limited to hindbrain and spinal commissural systems that rely on Netrin-mediated midline attraction. While we find that *Slit1* and *Slit2* transcript is present at the dorsal midline, *Netrin-1* transcript is absent, suggesting that DCNs do not rely on Netrin-mediated attraction to arrive at the dorsal midline. Further, ectopic expression of Robo3 in dorsal horn neurons does not elicit dorsal midline crossing (Escalante et al., 2013), consistent with our results and the view that Robo3 function may be limited to Netrin-dependent hindbrain and spinal commissural systems.

Formation of commissures in the forebrain also does not require Robo3 (Jen et al., 2004; Volk et al., 2011), despite ongoing requirement for Robo1, Robo2, and Slits (Ypsilanti et al., 2010). In further similarity to forebrain commissural populations, we have found that commissure formation at the dorsal midline occurs independently of a floor plate structure. Could alternative structures provide the cues required for DCN midline crossing? In the forebrain, commissure formation relies on transient midline glial populations to coordinate commissural neuron midline crossing (Lindwall et al., 2007; Chédotal and Richards, 2010). Moreover, perturbation of midline glial development, as in the *Nfia* mutant, has been shown to prevent midline crossing in multiple forebrain commissures (Shu et al., 2003; Lindwall et al., 2007). Radial glial fibers are present at the dorsal midline during DCN midline crossing (Comer and Kaltschmidt, unpublished observation), raising the possibility that midline glial-dependent mechanisms may also be used at the dorsal midline. However, while *Nfia* transcript expression is present at the dorsal midline during DCN midline crossing (Comer and Kaltschmidt, unpublilshed observation; Deneen et al., 2006), radial glia persist in the *Nfia* mutant and DCN midline crossing appears unchanged (Comer and Kaltschmidt, unpublished observation), indicating that other approaches are required to test the role of midline glia in coordinating dorsal commissure formation.

### DCNs and the Dorsal Midline

The dorsal midline itself may play a role in DCN commissure formation. DCN midline crossing in the rat and mouse similarly occur during the last quarter of embryogenesis, a period in which the dorsal midline morphologically changes as the mature form of the central canal emerges (Sturrock, 1981; Snow et al., 1990; Sevc et al., 2009). A critical aspect of this process appears to involve morphological changes in the roof plate as its structure changes from a wedge shape at earlier embryonic stages to a dorsal septum that extends from the pial surface to the dorsal aspect of the central canal (Sturrock, 1981; Snow et al., 1990; Kondrychyn et al., 2013). Interestingly, in the rat, during this period, the dorsal midline axon barrier composed of the glycosaminoglycan keratan sulfate is no longer detectable (Snow et al., 1990), which may make the dorsal midline more permissible for contralateral axonal growth. We considered whether the roof plate might play an attractive signaling role in formation of the dorsal commissure, analogous to the role of the floor plate in ventral commissure formation. However, our analysis of the *dreher* mutant, in which the roof plate does not form (Millonig et al., 2000), shows that this structure is not required for normal DCN midline crossing. Genetic targeting of other components of the dorsal midline may yet yield perturbations in DCN midline crossing.

### Nociceptive Modulatory Circuitry

Recent studies have improved our understanding of the neuronal circuitry underlying pain signaling in the spinal cord (Braz et al., 2014). Spinal interneurons in the dorsal horn, in particular, are felt indispensible for the inhibitory control of nociception (Sandkühler, 2009; Todd, 2010). Physiological studies of pain signaling have demonstrated the presence of contralateral inhibition (Fitzgerald, 1982), and unilateral nerve injury studies demonstrate changes in GABA immunoreactivity in the contralateral dorsal horn (Ibuki et al., 1997; Eaton et al., 1998). Both lines of evidence imply the involvement of inhibitory neurons that project contralaterally across the dorsal midline in pain signaling. The inhibitory spDCN population that we describe here is well suited to provide the anatomic basis for this contralateral inhibition. Nerve injury studies have demonstrated changes in sensory gene and protein expression in the contralateral dorsal horn following unilateral nerve manipulation (Wong and Oblinger, 1990; Zhang et al., 1996), suggesting that nociceptive sensory neurons may also project contralaterally across the dorsal midline. Our characterization of DCNs reveals such a population of sensory neurons and suggests that nociceptive snDCNs may similarly contribute to contralateral pain pathways. Given these observations, further characterization of the nature and connectivity of DCNs will contribute significantly to our understanding of the spinal circuitry of pain.

## Author Contributions

JDC performed experiments. FCP, SGW, and CVEW generated the *Ptf1a^Null/+^* mouse line. PH and KJM acquired samples for the *dreher* analysis. JDC and JAK designed the study, interpreted results, and wrote the paper.

## Conflict of Interest Statement

The authors declare that the research was conducted in the absence of any commercial or financial relationships that could be construed as a potential conflict of interest. The content of this study is solely the responsibility of the authors and does not necessarily represent the official views of the National Institutes of Health.
